# VCAN in the extracellular matrix drives glioma recurrence by enhancing cell proliferation and migration

**DOI:** 10.3389/fnins.2024.1501906

**Published:** 2024-11-01

**Authors:** Ruolun Wei, Haoyun Xie, Yukun Zhou, Xuhao Chen, Liwei Zhang, Brandon Bui, Xianzhi Liu

**Affiliations:** ^1^Department of Neurosurgery, The First Affiliated Hospital, Zhengzhou University, Zhengzhou, Henan, China; ^2^Department of Neurosurgery, School of Medicine, Stanford University, Stanford, CA, United States; ^3^Biochemistry and Molecular Biology, Georgetown University, Washington, DC, United States; ^4^Department of Veterinary Medicine, Huazhong Agricultural University, Wuhan, Hubei, China; ^5^Department of Pathophysiology, School of Medicine, Zhengzhou University, Zhengzhou, Henan, China; ^6^Department of Vascular Surgery, The First Affiliated Hospital, Zhengzhou University, Zhengzhou, Henan, China; ^7^Department of Human Biology, Stanford University, Stanford, CA, United States

**Keywords:** glioma, cancer recurrence, extracellular matrix, versican, PI3K/AKT pathway

## Abstract

**Introduction:**

Gliomas are the most prevalent primary malignant intracranial tumors, characterized by high rates of therapy resistance, recurrence, and mortality. A major factor contributing to the poor prognosis of gliomas is their ability to diffusely infiltrate surrounding and even distant brain tissues, rendering complete total resection almost impossible and leading to frequent recurrences. The extracellular matrix (ECM) plays a key role in the tumor microenvironment and may significantly influence glioma progression, recurrence, and therapeutic response.

**Methods:**

In this study, we first identified the ECM and the Versican (VCAN), a key ECM protein, as critical contributors to glioma recurrence through a comprehensive analysis of transcriptomic data comparing recurrent and primary gliomas. Using single-cell sequencing, we revealed heterogeneous distribution patterns and extensive intercellular communication among ECM components. External sequencing and immunohistochemical (IHC) staining further validated that VCAN is significantly upregulated in recurrent gliomas and is associated with poor patient outcomes.

**Results:**

Functional assays conducted in glioma cell lines overexpressing VCAN demonstrated that VCAN promotes cell proliferation and migration via the PI3K/Akt/AP-1 signaling pathway. Furthermore, inhibiting the PI3K/Akt pathway effectively blocked VCAN-mediated glioma progression.

**Conclusion:**

These findings provide valuable insights into the mechanisms underlying glioma recurrence and suggest that targeting both VCAN and the PI3K/Akt pathway could represent a promising therapeutic strategy for managing recurrent gliomas.

## Graphical abstract

Created with Biorender.com.

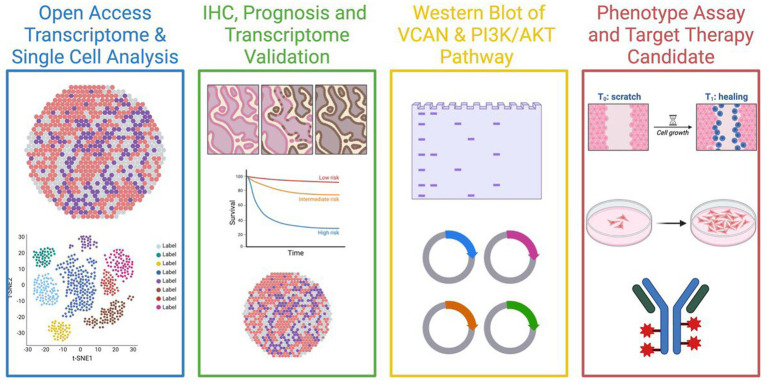

## Background

Gliomas are the most common primary intracranial tumors, accounting for approximately 81% of all malignant intracranial tumors (Lapointe et al., 2018). Although the incidence of gliomas is relatively low, representing only 2% of all primary cancers, their high rates of drug resistance, recurrence, and mortality make them a significant concern (Schaff and Mellinghoff, 2023). Histologically, gliomas exhibit substantial variability, ranging from benign ependymomas to highly aggressive and lethal grade IV glioblastoma (GBM). GBM is the most common histological form of glioma, constituting approximately 45% of all gliomas (Schaff and Mellinghoff, 2023). The 5-year survival rate for patients diagnosed with GBM is less than 6% (Wang and Wang, 2022). One of the main reasons for this poor prognosis is the diffuse infiltration of glioma cells (either individually or in clusters) into surrounding normal brain tissue, creating indistinct boundaries between the peripheral glioma layer and the surrounding normal brain tissue (Payne and Huang, 2013). Such infiltration complicates total resection, leading to the frequent recurrence of residual glioma cells at the original surgical site within months, forming tumors that are detectable both macroscopically and radiographically (Barbagallo et al., 2021). The high recurrence rate of gliomas and the limited treatment options present significant challenges for patients, highlighting the critical importance of research focused on glioma recurrence and the surrounding extracellular matrix (ECM) (Wei et al., 2024).

The extracellular matrix (ECM) is a complex stereoscopic structure composed of proteoglycans and fibrous proteins, surrounding and supporting the cells within the stroma (Theocharis et al., 2016). The intricate scaffold mainly provides a structural framework for cellular nesting. Once the structure skeleton is formed, the ECM further functions in regulating cellular processes such as proliferation, migration, differentiation, and apoptosis (Miller et al., 2020). ECM’s composition is dynamically adjusted depending on developmental stages, physiological environment and pathological conditions. For malignant tumors such as gliomas, ECM serves as a key component of the microenvironment, significantly influencing disease progression, metastasis, and therapeutic response.

Versican (VCAN) is a chondroitin sulfate proteoglycan produced by glioma cells, then secreted into the ECM, where it becomes a major component, playing a crucial role in the structure and function of the ECM (Timms and Maurice, 2020). VCAN is a complex molecule composed of glycosaminoglycan side chains and modular core protein domains, with various synthetic processes regulating these components (Wight et al., 2023). VCAN exhibits diverse spatiotemporal expression patterns across various cell types and during different developmental and pathological stages (Zhang et al., 2023). In various cancers, such as glioma and breast cancer, the VCAN expression level is upregulated and is often associated with poor prognosis, increased metastasis, and a higher likelihood of recurrence. These observations highlight the significant role of VCAN in the tumor microenvironment and its potential as a prognostic biomarker and therapeutic target.

Therefore, in this study, we focused on bioinformatic analysis of recurrent and primary glioma datasets and then the VCAN in the regulation of glioma cell invasion and migration to provide a new idea for the diagnosis and treatment of glioma recurrent.

## Method

### Data acquisition

Transcriptomic data, along with clinical and follow-up information, were obtained from the public glioma database, the Chinese Glioma Genome Atlas (CGGA)^1^ (Zhao et al., 2021). After excluding data with missing clinical information, we included 657 glioma samples for further analysis, comprising 372 males and 285 females.

### Differential expression genes identification

The differential expression genes (DEGs) cohort was assessed with the ‘limma’ R package (Ritchie et al., 2015). The thresholds of DEGs were as follows: The fold change (FC) of differential expression of mRNAs was |log2 fold change| ≥ 1 and False Discovery Rate (FDR) < 0.05 (Wight et al., 2020). DEGs list of recurrent vs. primary glioma patients was generated as ‘R/P_Diff’, DEGs list of long-term survival vs. non-long-term survival glioma patients was generated as ‘OS_Diff’, with the threshold set as 12 months. Cancer-recurrent associated genes were acquired from the ‘Genecard’ website and sorted as the gene list “Genecard_Recur.” The ‘Venn’ package is used to generate a Venn plot for the intersection check of the three lists.

### Function enrichment

Pathway enrichment of DEGs is using the Gene Ontology (GO) (Gene Ontology Consortium, 2015) and Kyoto Encyclopedia of Genes and Genomes (KEGG) (Kanehisa and Goto, 2000) databases with R packages “clusterprofiler,” and was further visualized by R package “ggplot2.” Gene expression levels were set as population phenotypes, and the related pathways and molecular mechanisms in GBM patients were evaluated using GSEA^2^ (Subramanian et al., 2005). Enriched gene sets with a nominal *p*-value of <0.05 and an FDR of <0.25 were considered statistically significant.

### PPI network

The STRING database^3^, an online tool for analyzing known and predicted protein–protein interaction (PPI) networks including both direct and indirect interactions and their functional correlations, was used to construct the PPI network (Szklarczyk et al., 2023).

### Signature construction

R packages ‘glmnet’ and ‘survival’ were used to perform Lasso-Cox regression analysis by integrating clinical data on patient overall survival (OS), survival status (Censor), and gene expression profiles, thereby minimizing the risk of overfitting. Multivariate Cox proportional hazard regression was used to develop the prognostic model, with patient risk scores calculated as follows:


$$\begin{array}{l} \mathrm{Risk}\;\mathrm{score}=\sum (\mathrm{expression}\;\mathrm{of}\;\mathrm{signature}\;\mathrm{genes}\\ \;*\mathrm{corresponding}\;\mathrm{coefficient})\text{.} \end{array}$$

Patients were assigned to high-risk by the top 25% of risk score rank or low-risk groups by the other 75% of risk score rank.

### Nomogram construction

A nomogram was created using the ‘rms’ package in R, with the upper section representing the scoring system and the lower section showing predictions. The total score, calculated by summing points for each factor, accurately predicted 1-, 3-, and 5-year survival and recurrence rates in glioma patients. Calibration curves and AUC were used to verify the predictive accuracy for overall survival (OS) and progression-free survival (PFS).

### Bayesian neural network gene orientation analysis

The “CBNplot” package, based on Bayesian neural networks, was employed to calculate the gene interaction network. The “bngeneplot” function was utilized to visualize the graph, using 0.95 as the filtering criterion strength (Sato et al., 2022).

### Single-cell data processing and analysis

Single-cell RNA sequencing (scRNA-seq) data were obtained from CGGA (CGGA.scRNA_6148), comprising a total of 6,148 cells from 73 regions in 14 glioma patients (Zhao et al., 2021). The analysis involved the following processing steps: (1) The R package “Seurat” was used to convert the 10× scRNA-seq data into Seurat objects; (2) data quality was assessed by examining the proportion of mitochondrial and ribosomal genes, with low-quality cells excluded from further analysis; (3) following quality control, the “FindVariableFeatures” function was applied to identify the top 2,000 highly variable genes; (4) principal component analysis (PCA) was conducted using these 2,000 genes, followed by dimensionality reduction and cluster identification using t-distributed stochastic neighbor embedding (t-SNE); (5) differential gene markers for each cluster were identified using the “Find All Markers” function, with a log2 [fold change (FC)] threshold set to 0.3 and a minimum percentage threshold of 0.25; (6) the “SingleR” package was used to annotate clusters and classify cell types; (7) intercellular communication networks were inferred and analyzed using the tools “CellChat” and “scMLnet,” focusing on receptor-ligand interactions.

### Patient and samples

The collection of samples and clinical information in this study was approved by the Ethics Committee of the First Affiliated Hospital of Zhengzhou University, and written informed consent was obtained from each patient. Each sample was examined and diagnosed by at least two neuropathologists as diffuse glioma (WHO grades II-IV). Isocitrate dehydrogenase (IDH) mutation and 1p/19q codeletion status were determined by the pathology department. Patients were followed up every 3 months through phone calls or clinical visits. At the follow-up endpoint of the study, the average survival time for the cohort was 687.9 days, with 19 patients censored and 42 patients still alive.

### Immunohistochemistry (IHC)

Paraffin-embedded glioma tissue sections were deparaffinized, hydrated, and antigen retrieval was conducted using a microwave with citrate buffer (pH 9.0). To block endogenous peroxidase activity, sections were incubated in 0.3% H₂O₂ for 10 min. The sections were then treated with primary antibody at 4°C for 12 h, followed by incubation with secondary antibody at room temperature for 1 h. After staining with peroxidase and DAB, sections were counterstained with hematoxylin and mounted on a nonaqueous medium. Images were captured using the KF-PRO-020 digital slice scanner, and two independent pathologists evaluated and scored the samples. VCAN expression was detected using an anti-Versican antibody (1:200, ABclonal, A19655).

### Cell line and culture

Human astrocytes (HA1800) and glioma cell lines (U87-MG, LN229) were obtained from the Stem Cell Experimental Center of Zhengzhou University. Cells were cultured in Dulbecco’s modified Eagle medium (DMEM; Hyclone, UT, USA) supplemented with 10% fetal bovine serum (FBS; Thermo Fisher Scientific, MA, USA) and maintained at 37°C in a humidified 5% CO₂ environment.

### Cell transfection

Overexpression (OE) and normal control (NC, shNC) lentivirus were purchased from Genechem (Shanghai, China). The transfected glioma cells (U87-MG, LN229) were cultured in a puromycin-containing medium, the stable cell was selected with 2.5 μg/mL of puromycin, and the GFP fluorescence intensity of cells was observed daily using fluorescence microscopy until no significant change in fluorescence intensity was noted, and no dead floating cells were present. Overexpression of VCAN in the U87-MG and LN229 cell lines was achieved using Lipofectamine 2,000 to deliver the plasmid. The efficacy of transfection was confirmed through western blot. Control glioma cells were transfected with the empty vector.

### Western blot

Total proteins were extracted using RIPA lysis buffer (ThermoFisher, USA) with protease and phosphatase inhibitors (MCE) and incubated on ice for 15 min. After centrifugation (12,000 × g, 4°C, 2 min), supernatants were collected, and protein concentration was measured with a BCA protein assay kit (ThermoFisher, USA). Denatured proteins were separated on 10% SDS-PAGE gels (Bio-Rad), transferred to 0.2 μm PVDF membranes (Millipore), and blocked with skimmed milk for 1 h. Membranes were cut, with adjacent two markers saved in one gel strip. The gel strip was incubated with primary antibodies at 4°C for 15 h, followed by secondary antibodies at room temperature for 1 h. After washing in TBST, the membranes were visualization.

ImageJ software was used to analyze the grayscale values of the bands for quantification.

### Colony formation assay

Cells were treated with vehicle control, overexpression plasmid or overexpression plasmid with an inhibitor for 48 h. After trypsinization and counting, 2000 cells per well were plated in 6-well dishes and incubated for 10 days. The medium was refreshed every 3 days. Colonies were fixed with 10% formaldehyde for 30 min and stained with 0.5% crystal violet. Colonies containing over 50 cells were counted under a microscope, and the percentage of cell survival was calculated. The experiment was repeated in triplicate.

### Wound healing assay

To assess cell migration, 1 × 10^5^ glioma cells were seeded in 6-well plates. Upon reaching 90% confluency, a sterile 200-μL pipette tip was used to create a scratch. Cells were washed with PBS and treated with serum-free DMEM containing vehicle control (DMSO) or the overexpression plasmid for 48 h. Images were captured at 0 h and 36 h, and the wound healing area was quantified using ImageJ software. Each condition was performed in triplicate.

### Dose–response assay

Cells were seeded at 5,000 cells per well in 96-well plates and incubated with or without treatment for 3 days at 37°C. Cell viability was assessed using the CellTiter-Glo Luminescent Cell Viability Assay (Promega) according to the manufacturer’s instructions. Luminescence was measured using a BioTek Synergy HTX reader. Data represent the mean ± standard deviation for three independent replicates.

### Statistical analysis

Pearson’s or Spearman’s coefficients were used to analyze correlations between variables. R language v4.0.2^4^ and GraphPad Prism 8.0 software were conducted for data analysis. Quantitative data were compared using an independent *t*-test for two samples and a one-way ANOVA for multiple samples. These results were considered statistically significant at *p < 0.05, **p < 0.01, ***p < 0.001, and ****p < 0.0001.

## Results

### ECM actively involves in glioma recurrent

**Figure 1:**
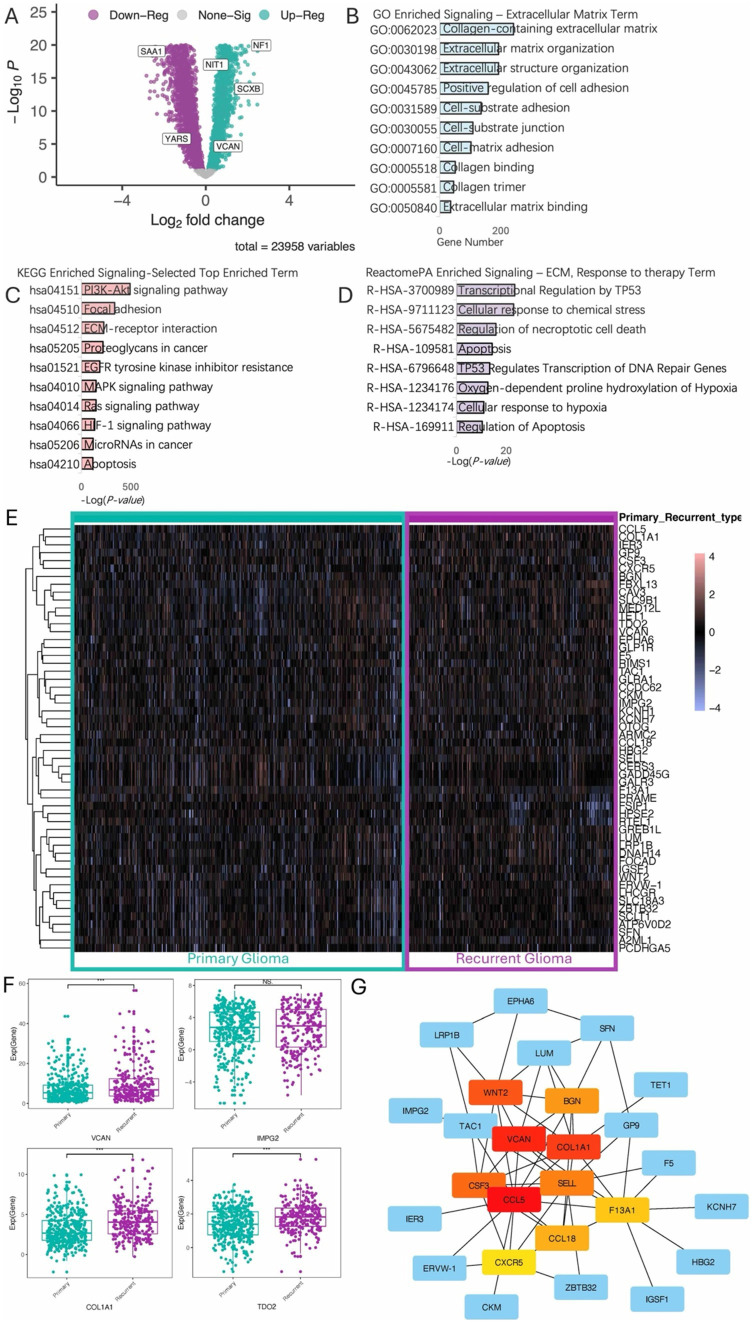
Differential analysis of recurrent and primary gliomas. **(A)** Volcano plot illustrating differentially expressed genes between recurrent and primary glioma cohorts. **(B)** Gene Ontology (GO) enrichment analysis highlighting terms associated with the ECM. **(C)** KEGG (Kyoto Encyclopedia of Genes and Genomes) enrichment analysis, displaying the top enriched terms. **(D)** ReactomePA enrichment analysis, displaying ECM-related and response to therapy pathways. **(E)** Heatmap of the top 50 differential expression genes. **(F)** Boxplot shows upregulation of specific genes in the recurrent glioma cohort. **(G)** Hierarchical analysis of protein–protein interaction (ppi) network.

Analysis of Differentially Expressed Genes (DEGs) revealed significant upregulation of several genes in recurrent glioma, including NF1, NIT1, SCXB, and VCAN (Figure 1A). Functional enrichment analysis using Gene Ontology (GO) (Figure 1B), KEGG pathways (Figure 1C), and ReactomePA (Figure 1D) indicated that these DEGs are highly involved in ECM functions, proteoglycans, collagens, and cell junctions. By intersecting gene groups associated with cancer recurrence, differential expression between primary and recurrent gliomas, prognosis-related genes, and pan-cancer recurrence-related genes using a Venn diagram (Supplementary Figure 1), we identified 54 key DEGs (Supplementary Table 1). These genes exhibited distinct expression patterns between recurrent and primary gliomas (Figure 1E). Notably, several classic ECM-related genes were significantly upregulated in the recurrent cohort, consistent with the pathway enrichment results (Figure 1F). Further analysis of these 54 genes through protein–protein interaction (PPI) network analysis, excluding isolated nodes, revealed a closely interacting network of DEGs in recurrent gliomas, prominently featuring genes such as WNT2, BGN, COLIAI, and VCAN (Figure 1G).

### Recurrent-based RAEM score reliably stratifies patients into prognostic groups

**Figure 2:**
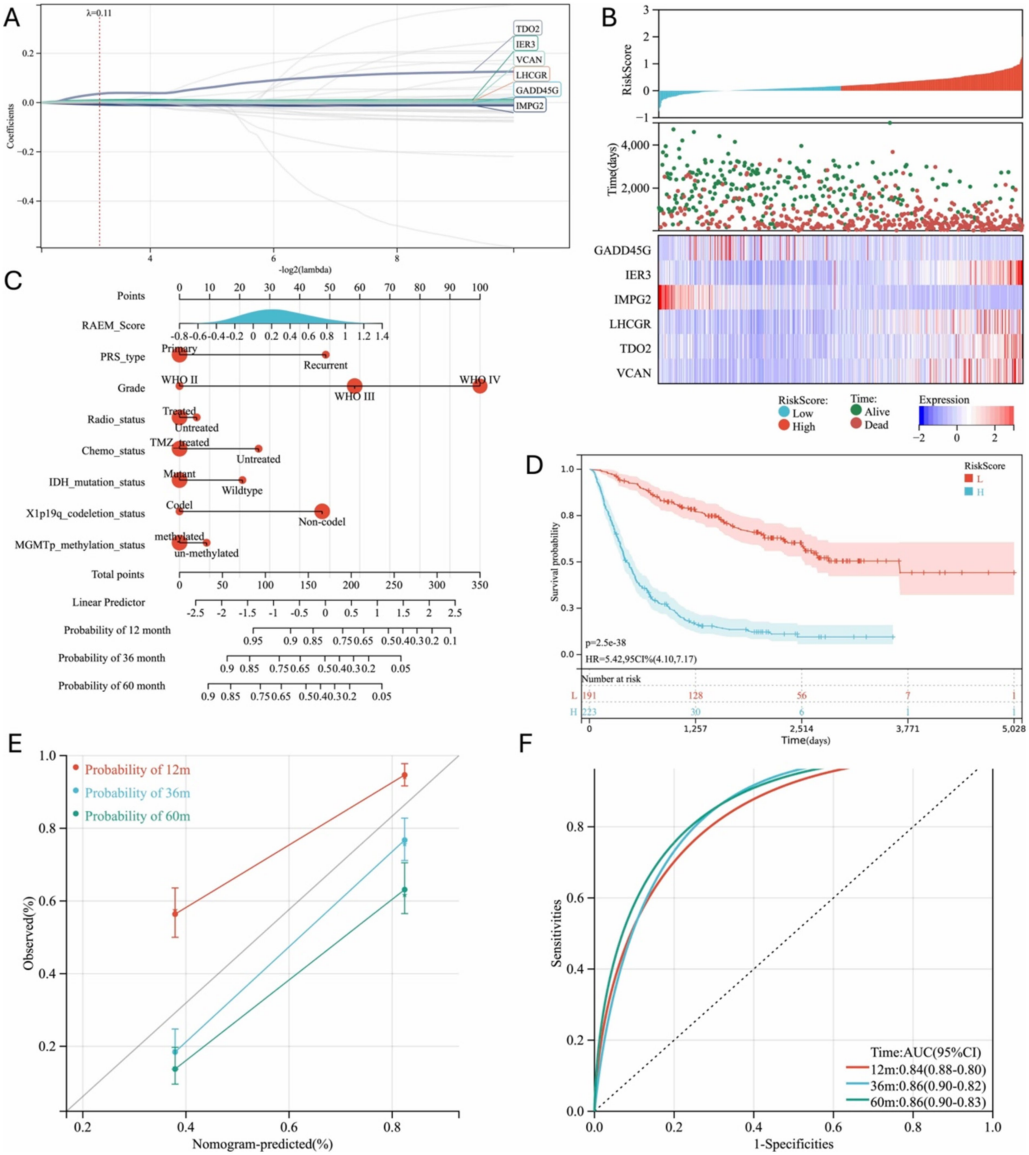
Regression analysis of DEGs and progression model generated. **(A)** Six independent risk genes were screened out by Lasso-Cox to establish a regression model; **(B)** Prognosis and expression heat map of six independent risk genes; **(C)** Nomogram used to predict gene expression-related recurrence probability in glioma patients; **(D)** Kaplan–Meier survival curve of different groups based on RAEM risk score, 12 m: 12-month, 36 m: 36 month, 60 m: 60-month; **(E)** calibration chart of 1-year, 3-year and 5-year RFS predicted by Cox regression model; **(F)** ROC curve and area under the curve (AUC) of the prediction model, 12 m: 12-month, 36 m: 36 month, 60 m: 60-month.

Following the closely interacting network of DEGs, we further investigated the core functional genes using Lasso-Cox and multivariate Cox regression analyses. This analysis identified six independent prognostic factors (Figure 2A), leading to the formulation of the risk score model:


$$\begin{array}{l} \mathrm{Risk}\;\mathrm{score}=\mathrm{exprGADD}45G\;x\;\mathrm{\lambda GADD}45G\\ \;+\mathrm{exprIER}3\;x\;\mathrm{\lambda IER}3+\mathrm{exprIMPG}2\;x\;\mathrm{\lambda IMPG}2\\ \;+\mathrm{exprLHCGR}\;x\;\mathrm{\lambda LHCGR}+\mathrm{exprTDO}2\;x\;\mathrm{\lambda TDO}2\\ \;+\mathrm{exprVCAN}\;x\;\mathrm{\lambda VCAN}\text{,} \end{array}$$

where expr*GENE* represents the expression level of each gene, and λ*GENE* is the corresponding coefficient. Using 10-fold cross-validation, the optimal model was determined with a lambda value of 0.110422641992181 (Figure 2B). The selected genes and their corresponding lambda coefficients are as follows: GADD45G: −1.87276819926125e-05, IER3: 0.00869098797806007, IMPG2: -0.00798186119301403, LHCGR: 0.0010179392653652, TDO2: 0.0356031707508411, and VCAN: 0.00467325885111677. This culminated in the Recurrent Associated Expressed Molecules (RAEM) score, defined as:


$$\begin{array}{l} \mathrm{RAEM}\_\mathrm{RiskScore}=-1.87276819926125e-05\\ \;*\mathrm{GADD}45G+0.00869098797806007*IER3\\ \;-0.00798186119301403*\mathrm{IMPG}2+0.0010179392653652\\ \;*\mathrm{LHCGR}+0.0356031707508411*TDO2\\ \;+0.00467325885111677*\mathrm{VCAN}\text{.} \end{array}$$

A heatmap of gene expression and patient prognosis shows as survival time decreases and prognosis worsens, the expression of IER3, LHCGR, TDO2, and VCAN (risk factors) progressively increases, while the expression of GADD45G and IMPG2 (protective factors) gradually decreases (Figure 2C). Patients in the top 25% of RAEM scores were classified as the high-risk group, while the remainder were considered the low-risk group. Kaplan–Meier survival curves demonstrated that the RAEM score effectively stratifies patients by prognosis (Figure 2D). The RAEM score, along with other influential factors, is visualized in a nomogram (Figure 2E). The nomogram predictions showed excellent consistency with actual observations for 1-year, 3-year, and 5-year survival rates (Figure 2F) and demonstrated strong predictive ability for 1-year, 3-year, and 5-year OS rates (Figure 2G).

### RAEM score represents cluster signature

**Figure 3:**
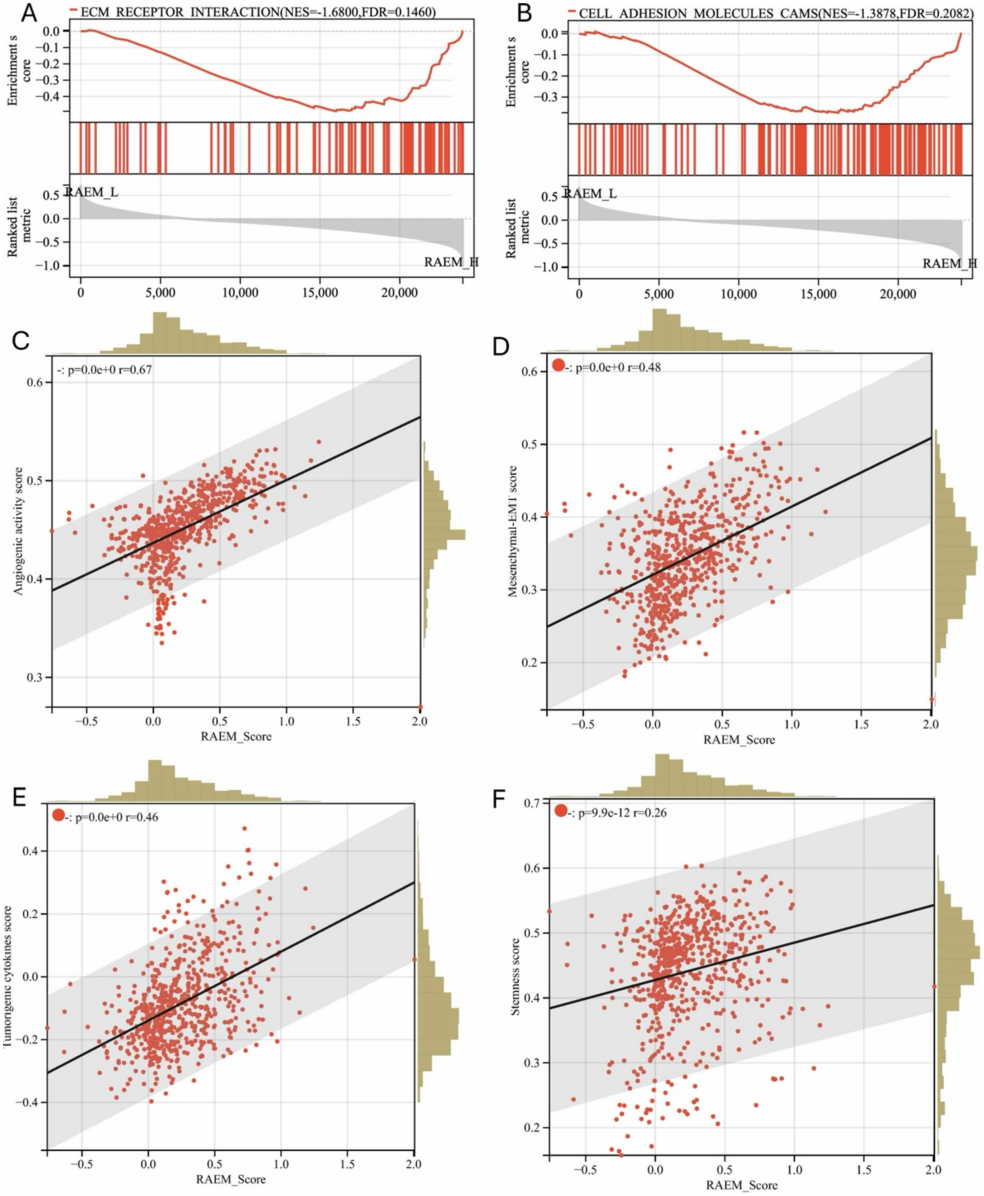
GSEA functional enrichment and estimate regression analysis of recurrent-associated expressed molecules (RAEM). GSEA enrichment pathway shows **(A)** ‘ECM receptor binding’ and **(B)** ‘CAMS: cell adhesion molecules’ are significantly upregulated (FDR < 0.25, *p* < 0.05); **(C)** regression analysis of RAEM score and ESTIMATE algorithm, showing the correlation between RAEM score and **(C)** angiogenic activity index, **(D)** epithelial-mesenchymal transition index, **(E)** tumorigenic cytokine index; and **(F)** stemness index have linear positive correlation, *p* < 0.05.

Gene Set Enrichment Analysis (GSEA) was conducted on the CGGA expression profile based on the RAEM score. Several components of the tumor microenvironment and ECM, including ECM-receptor interactions (Figure 3A) and cell adhesion molecule aggregation (CAMs) (Figure 3B), were highly correlated with the RAEM score. The ESTIMATE algorithm analysis, based on the ratio of immune and stromal cells, showed that the RAEM high-risk group exhibited higher angiogenesis activity (Figure 3C), EMT index (Figure 3D), tumorigenic cytokine index (Figure 3E), and stemness index (Figure 3F) compared to the low-risk group. A linear correlation was observed, potentially confirming the association between the RAEM score and high recurrence potential.

### Single-cell analysis unveils extensive communication within ECM networks

**Figure 4:**
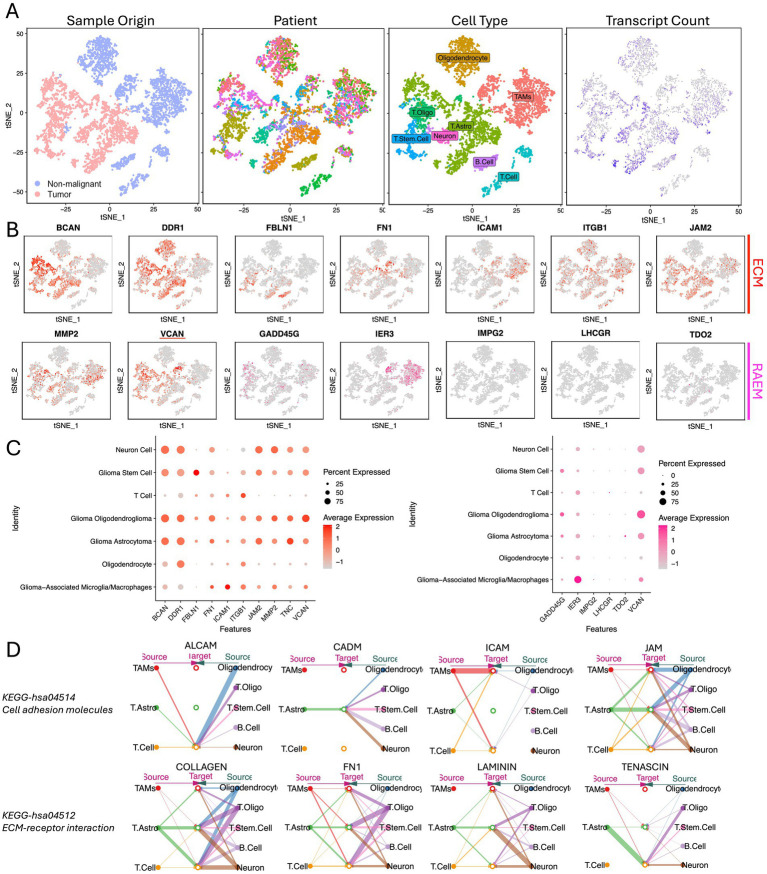
Single-cell expression atlas of human glioma from CGGA. **(A)** tSNE of the single-cell sequencing profiled here, with each cell color-coded for (left to right): sample type of origin (tumor or non-malignant cell), the corresponding patient, the associated cell type, and the number of transcripts (UMIs) detected in that cell (log scale as defined in the inset). Legend for patient sample figure is shown in supplemental file. **(B)** Expression of ECM and RAEM marker genes for the cell types defined above each panel. **(C)** Percentage expression of ECM and RAEM marker genes among cell types. **(D)** Intercellular communication networks of ECM components.

Single-cell RNA-Seq analysis was conducted using CGGA open-access data. Utilizing ‘SingleR’ for automated annotation and differential gene expression analysis, we first identified the malignant cell populations. Subsequently, we characterized the composition of cell populations, identifying eight distinct cell subpopulations: oligodendrocyte, tumor-associated macrophages (TAMs), oligodendrocytoma tumor (T oligo), astrocytoma tumor (T astro), tumor stem cell (T stem cell), neuron, B-cells and T-cells (Figure 4A). ECM components exhibited differential distribution across these subpopulations. Notably, BCAN, VCAN, and JAM2 were predominantly expressed by tumor cells, while ITGB1 was ubiquitously expressed across all clusters. ICAM1 was highly upregulated in TAMs, but DDR1 was expressed across all subpopulations except TAMs (Figures 4B,C). Then we identified the ligand-receptor interactions among cell groups and ECM components using ‘CellChat’. CellChat emphasizes the significant role of ECM-related ligand-receptor pairings in glioma. The key pathways of ECM function involved in ligand-receptor interactions were identified as KEGG-hsa04514 (cell adhesion molecules pathway) and KEGG-hsa04512 (ECM-receptor interaction), reinforcing the critical role of stromal-based interactions in glioma progression (Figure 4D).

### VCAN is the key factor acting in the functioning ECM network

**Figure 5:**
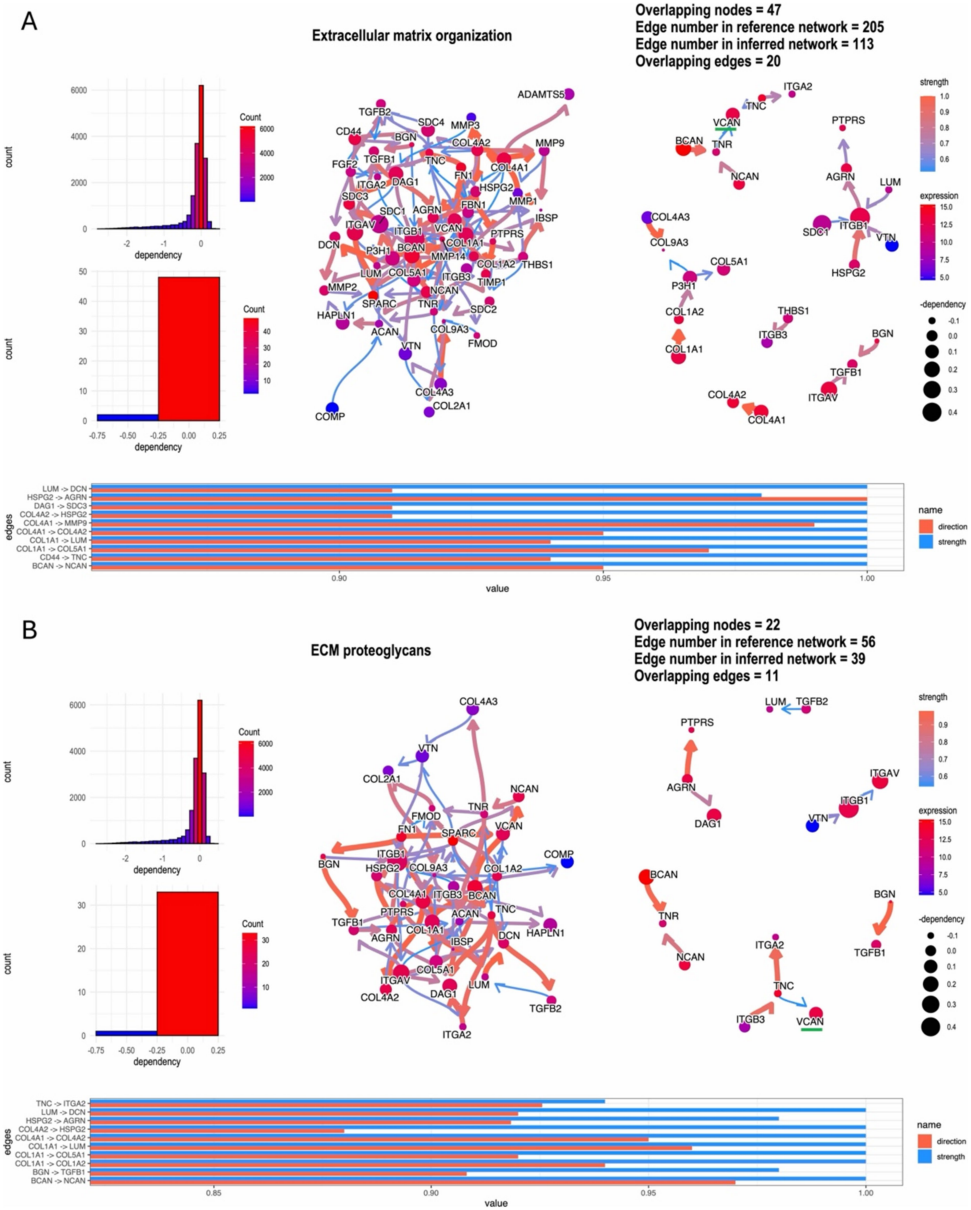
‘CBNplot’ Bayesian gene regulatory network analysis, **(A)** the most highly enriched network: ECM programming; **(B)** the second highly enriched network: ECM proteoglycans.

Using the Bayesian network package CBNplot for gene regulation causal analysis, the most highly enriched network was ECM programming, with 47 enriched node genes, including highly expressed VCAN, BCAN, ITGB1, and COL4A1 (Figure 5A). The second most enriched network was ECM proteoglycans, containing 22 enriched node genes, with VCAN, ITGA5, and DAG1 highly expressed (Figure 5B). These analyses indicate that the ECM is crucial in glioma recurrence and progression. VCAN, as a highly expressed node gene in both networks, may be pivotal for glioma recurrence and progression.

### Glioma VCAN expression upregulated when recurrence and associated with poor patient prognosis

**Figure 6:**
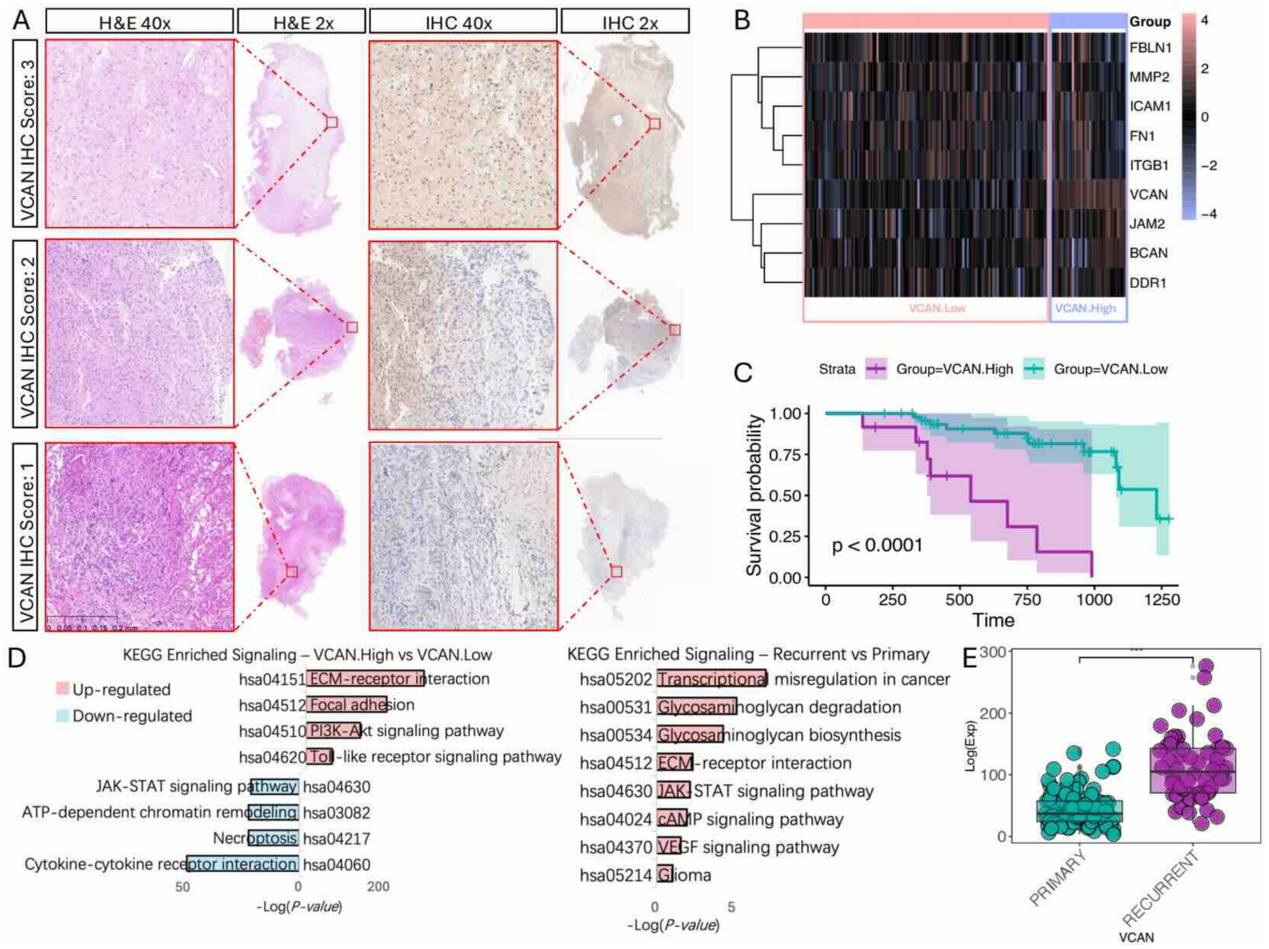
Immunohistochemistry and bulk-RNA transcriptome validation of VCAN expression and clinical significance. **(A)** VCAN-IHC staining of glioma sample with representative VCAN-IHC staining of score 3 (strong staining), score 2 (moderate staining), and score 1 (weak staining) group, with corresponding H&E stains. **(B)** Heatmap of ECM components gene expression level with different VCAN level groups. **(C)** Kaplan Meier survival analysis of different VCAN staining score groups. **(D)** KEGG analysis on different VCAN levels and recurrent/primary states. **(E)** VCAN expression level in primary/recurrent glioma.

To validate these findings, we conducted bulk RNA sequencing and transcriptome analysis on 150 glioma specimens obtained from the Department of Neurosurgery at the First Affiliated Hospital of Zhengzhou University (Table 1). Among these specimens, formalin-fixed paraffin-embedded (FFPE) slides from 61 patients were acquired and subjected to hematoxylin and eosin (H&E) staining and VCAN immunohistochemical (IHC) analysis (Figure 6A). In the IHC evaluation, 12 patients had a VCAN-IHC score of 3, 32 had a score of 2, 17 had a score of 1, and none of the patients received a score of 0. Patients with a VCAN-IHC score of 3 were categorized as VCAN.High, while those with scores of 1 or 2 were classified as VCAN.Low. For the transcriptome analysis, patients were ranked according to VCAN expression levels, with the top 25% categorized as VCAN.High and the remaining patients as VCAN.Low. When comparing the high VCAN expression group with the low VCAN expression group, the results showed that as VCAN expression increased, other ECM components such as FBLN1, MMP2, and BCAN were also upregulated in a coordinated manner. This finding aligns with the results from the Bayesian network analysis, which identified VCAN as a central gene in the ECM regulatory network (Figure 6B). Survival analysis revealed that the low-risk group (VCAN-IHC scores of 1 and 2) exhibited significantly longer survival times and better prognoses compared to the high-risk group (*p* < 0.05) (Figure 6C). KEGG pathway analysis demonstrated that, compared to the VCAN.Low group, the VCAN.High group exhibited upregulation of ECM-related functions, cell adhesion, and the PI3K/Akt and Toll-like receptor pathways while downregulating cytokine–cytokine receptor interaction. Additionally, KEGG analysis of recurrent versus primary gliomas revealed pathways similar to those observed in the CGGA dataset, further confirming that VCAN is upregulated in recurrent gliomas and could serve as a key factor in glioma recurrence (Figure 6D).

**Table 1:** Summary of bulk-RNA transcriptome from the First Affiliated Hospital of Zhengzhou University.

Characteristics	All patients, *N* = 150
Age, years	
Average	49.38
Range	18–78
CNS WHO Grade, *n* (%)	
II	39 (26.0%)
III	13 (8.7%)
IV	98 (65.3%)
Histology, *n* (%)	
Glioblastoma	93 (62.0%)
Oligodendroglioma	24 (16.0%)
Astrocytoma	33 (22.0%)
IDH, *n* (%)	
Mutant	57 (38.0%)
Wild	93 (62.0%)
1p/19q, *n* (%)	
Co-deletion	24 (16.0%)
Non co-deletion	126 (84.0%)
P/R State, *n* (%)	
Primary	80 (53.3%)
Recurrent	70 (46.7%)

### VCAN regulates glioma behavior through the PI3K/Akt pathway

**Figure 7:**
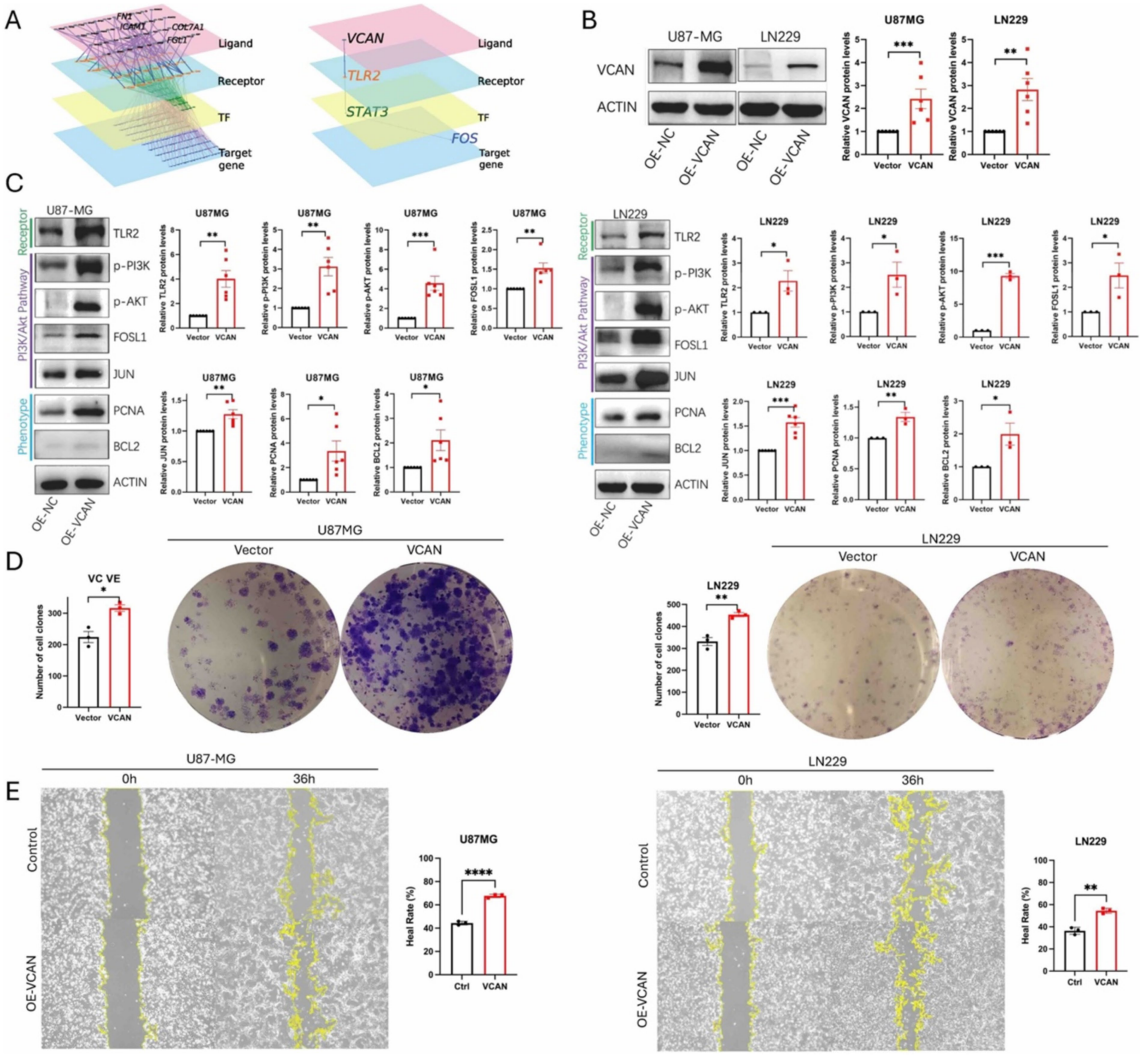
**(A)** scMLnet ligand-receptor network analysis shows TLR2 is the first downstream receptor of ligand VCAN. **(B)** Construction of VCAN overexpression-stable transfection U87MG and LN229 glioma cell lines. **(C)** Immunoblotting analysis of the protein expression levels of receptor TLR2, PI3K/AKT signaling pathway proteins p-PI3K, p-AKT, FOSLI, JUN and phenotype proteins PCNA and BCL2 in VCAN overexpression and control glioma U87MG and LN229 cell lines. Protein levels were quantified by the indicated band intensities. ACTIN was used as a normalization control for western blotting. **(D)** Plate cloning experiments verified the differences in the proliferation ability between VCAN overexpression and control U87-MG cells and LN229 cells. **(E)** Wound healing assay verified the differences in the migration ability between VCAN overexpression and control U87-MG cells and LN229 cells. Data were repeated three times. *p* < 0.05, **p < 0.01, and ***p < 0. 001.

Given that KEGG analysis of both the CGGA and our dataset indicates upregulation of the PI3K/Akt pathway in the high VCAN group, and single-cell analysis reveals extensive intercellular communication initiated by ECM components, we sought to investigate whether VCAN regulates glioma cell epigenetic behavior through the PI3K/Akt pathway. First, we utilized scMLnet to reconstruct the signaling network. Through analysis of multiple ligand-receptor, receptor-transcription factor (TF), and TF-target gene interactions, TLR2 emerged as the receptor through which VCAN functions as a ligand (Figure 7A). We then constructed VCAN overexpression stable transfection U87MG and LN229 glioma cell lines (Figure 7B). With VCAN overexpression, we observed upregulation of its receptor TLR2 and key proteins in the PI3K/Akt pathway, including p-PI3K, p-Akt, FOSL1, and JUN. FOSL1 and JUN are the downstream factors in the PI3K/Akt pathway, and they can form a stable heterodimer known as AP-1, a transcriptional activator that can influence glioma behavior, reflected by elevated levels of phenotype markers BCL2 and PCNA. Phenotypic assays, including colony formation (Figure 7D) and wound healing (Figure 7E), further demonstrated that VCAN overexpression enhances glioma cell proliferation and migration.

### Inhibition of VCAN-mediated glioma progression by targeting the PI3K/Akt pathway

**Figure 8:**
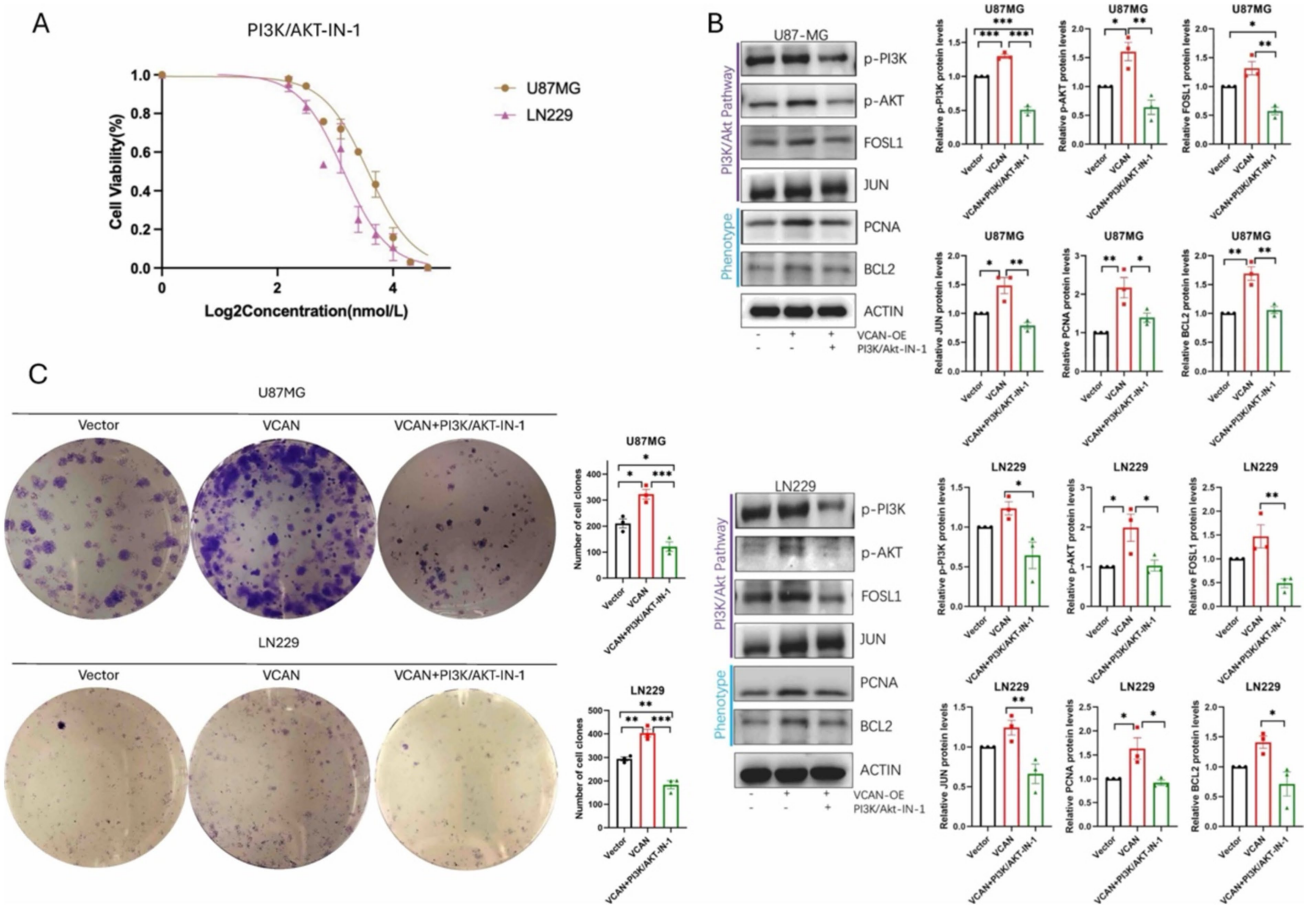
PI3K/AKT-IN-1 on blocking VCAN-mediated glioma progression by inhibiting the PI3K/Akt pathway. **(A)** Drug response of PI3K/AKT-IN-1 on U87MG and LN229, with IC50 of 2.2uM and 1.8uM. **(B)** Immunoblotting analysis of the protein expression levels of PI3K/AKT signaling pathway proteins p-PI3K, p-AKT, FOSLI, JUN and phenotype proteins PCNA and BCL2 in control, VCAN overexpression and VCAN overexpression with PI3K/AKT-IN-1 glioma U87MG and LN229 cell lines. Protein levels were quantified by the indicated band intensities. ACTIN was used as a normalization control for western blotting. **(C)** Plate cloning experiments verified the differences in the proliferation ability between control, VCAN overexpression and VCAN overexpression with PI3K/AKT-IN-1 glioma U87MG and LN229 cell lines. Data were repeated three times. *p* < 0.05, **p < 0.01, and ***p < 0. 001.

Given that VCAN regulates the AP-1 transcriptional activator, thereby influencing cell proliferation and migration, we explored the possibility of disrupting VCAN-mediated glioma progression by directly inhibiting the PI3K/Akt pathway, as no specific inhibitor for VCAN currently exists. To this end, we applied PI3K/AKT-IN-1, a PI3K-Akt signaling pathway inhibitor, to assess its impact on blocking VCAN-mediated upregulation of AP-1 and the subsequent effects on phenotype proteins and cell proliferation. Dose–response analysis revealed that PI3K-IN-1 exhibited IC50 values of 2.2 μM and 1.8 μM in U87MG and LN229 cell lines, respectively (Figure 8A). The inhibitor effectively interrupted the PI3K/Akt pathway and AP-1 protein upregulation and significantly suppressed the VCAN-induced enhancement of cell proliferation. These findings suggest that targeting the PI3K/Akt pathway could serve as a therapeutic strategy to counteract VCAN-induced glioma recurrence (Figures 8B,C).

## Discussion

In this study, we identified the key role of the ECM protein VCAN in the process of glioma recurrence. Our findings demonstrate that VCAN expression is elevated in recurrent gliomas and is associated with poor prognosis. We also provide evidence that VCAN contributes to the proliferation and migration of glioma cells.

Recurrent growth occurs in most cases of malignant glioma (Barthel et al., 2019). Following initial resection, the treatment of gliomas often involves chemotherapy, radiotherapy, and immunotherapy (Wen et al., 2020). However, nearly all malignant gliomas undergo recurrence, with 75 to 90% of recurrences occurring within 2 cm of the initial tumor margin, highlighting the importance of studying the ECM in both local control and global control (Obiri-Yeboah et al., 2023). Recurrent gliomas are often more drug-resistant than primary tumors due to factors such as a more heterogeneous and adaptive tumor microenvironment (Wu et al., 2023). The overexpression of ECM proteins such as VCAN enhances tumor cell proliferation, migration, and chemotherapy resistance by creating a physical barrier that impedes drug penetration. Additionally, recurrent tumors tend to accumulate genetic mutations and epigenetic changes that contribute to therapy resistance, making them more difficult to treat (Barthel et al., 2022). Recurrent gliomas are often more drug-resistant than primary ones due to several factors, including the presence of a more heterogeneous and adaptive tumor microenvironment, which is enhanced by changes in the ECM. The ECM, particularly the overexpression of proteins such as VCAN, contributes to increased tumor cell proliferation, migration, and protection from chemotherapy by creating a physical barrier that impedes drug penetration (Cheng et al., 2020). Additionally, recurrent tumors tend to accumulate genetic mutations and epigenetic alterations that confer resistance to therapies that were initially effective. This evolutionary process, driven by selective pressure from initial treatments, results in a population of tumor cells that are more resilient and adaptable, making them harder to treat in subsequent recurrences.

Differentially expressed genes were used to establish recurrence correlation analysis expression coefficients, which can accurately predict glioma recurrence and prognosis. Bayesian gene regulatory network analysis further indicated significant ECM enrichment in recurrent gliomas, with VCAN emerging as a key gene in this pathway. Single-cell sequencing analysis confirmed high VCAN expression in tumor-related cell subpopulations within heterogeneous glioma cell populations. Collectively, these findings suggest that ECM reorganization, with VCAN as a central player, is a key pathway in glioma recurrence.

Limited information is available on the role of the ECM, particularly VCAN, in glioma, especially in the context of tumor recurrence. VCAN is a large chondroitin sulfate proteoglycan with an apparent molecular mass of more than 1,000 kDa (Islam and Watanabe, 2020). In adults, this proteoglycan serves as a structural macromolecule of the ECM in the brain and large blood vessels. Studies have highlighted VCAN’s role in tissue morphogenesis and organogenesis, with its highest expression observed in the lung, liver, heart, and brain at embryonic day E13, when VCAN fragments remain low (Snyder et al., 2015). VCAN also plays a role in early-stage inflammation, where infiltrating inflammatory cells secrete proteinases and cytokines/chemokines (Wight et al., 2020). These processes degrade the ECM and activate stromal fibroblasts and endothelial cells, contributing to tissue repair and granulation. VCAN is expressed in activated fibroblasts, endothelial cells, and infiltrating macrophages, linking inflammation with tumor development and progression (Ball et al., 2016). Evidence shows that VCAN is involved in both malignant transformation and tumor progression. Its elevated expression has been reported in various malignancies, including liver and prostate cancers, and is associated with tumor recurrence and poor prognosis (Xia et al., 2014; Arichi et al., 2015; Voutilainen et al., 2003).

Our research revealed ECM reorganization, with VCAN as a central player, is a key pathway in glioma recurrence. VCAN, as a TLR2 ligand, can further activate the downstream PI3K/Akt pathway, ultimately leading to the activation of the AP-1 transcription factor, which promotes glioma progression (Hope et al., 2016). Although our research and other studies suggest that VCAN is a promising therapeutic target, no specific treatments have been developed yet. This may be due to the fact that VCAN is a critical component of the ECM and is also present in small amounts in normal tissues. Given that VCAN operates through the PI3K/Akt pathway, we hypothesized that direct inhibition of this pathway could block VCAN-induced cell proliferation. PI3K/Akt-IN-1 effectively inhibited the pathway, suppressed AP-1 protein, and reduced proliferation driven by VCAN overexpression, indicating that this approach could potentially prevent glioma recurrence on a larger scale.

Targeting ECM components, such as VCAN, may represent a promising therapeutic approach for glioma. Cilengitide, a selective inhibitor targeting integrins αvβ3 and αvβ5 in the ECM, regulates cell adhesion (Reardon et al., 2008). Preclinical studies have demonstrated that cilengitide exhibits enhanced antitumor effects when administered as part of combinatorial therapeutic regimens (Mikkelsen et al., 2009). However, the research was discontinued at the phase III clinical trial stage. Another drug, Neuradiab, specifically targets the ECM component Tenascin and is currently being investigated in a phase II clinical trial for the treatment of recurrent gliomas (Shimizu et al., 2016). At present, targeting the ECM appears to be a promising therapeutic approach. However, the key to this type of targeted therapy may lie in the method of local drug delivery. Previous clinical trials with Cilengitide have highlighted the potential of local delivery, which could be a crucial factor in targeting ECM components. Although ECM components are highly expressed in tumors, they are also present in normal tissues, meaning systemic administration could lead to off-target side effects. Therefore, localized delivery methods targeting ECM components may present a hopeful avenue for treatment.

In conclusion, VCAN is upregulated in recurrent gliomas and plays a key role in regulating glioma cell proliferation and migration via the PI3K/Akt/AP-1 pathway. The elevated expression of VCAN in recurrent gliomas suggests that it may confer a protective advantage to glioma cells in primary tumors, contributing to treatment resistance and ultimately facilitating recurrence. Future research by our group aims to further elucidate these mechanisms, with the objective of targeting VCAN degradation as a novel therapeutic strategy for the treatment of recurrent gliomas.

## Data Availability

The sequencing data can be obtained at the Open Archive for Miscellaneous Data (https://ngdc.cncb.ac.cn/omix/) of National Genomics Data Center (NGDC) with the accession no. OMIX007703.

## Footnotes

^1^http://www.cgga.org.cn

^2^http://software.broadinstitute.org/gsea/index.jsp

^3^https://string-db.org/

^4^https://www.r-project.org/
