# Supplementary material for: VCAN in the extracellular matrix drives glioma recurrence by enhancing cell proliferation and migration
Source: Front Neurosci. 2024 Nov 1;18:1501906. doi: 10.3389/fnins.2024.1501906 (PMC11565936; doi:10.3389/fnins.2024.1501906)
Supplement: Supplementary file 1 [file Data_Sheet_1.DOCX]

Supplemental File

**Supplemental Table 1** 54 genes in the intersection of Genecard cancer recurrence-related genes, glioma primary/recurrence differentially expressed genes and glioma survival difference-related genes

| A2ML1 | ARMC2 | ATP6V0D2 | BGN | CAV3 |
| --- | --- | --- | --- | --- |
| CCDC62 | CCL18 | CCL5 | CERS3 | CKM |
| COL1A1 | CSF3 | CXCR5 | DNAH14 | EPHA6 |
| ERVW-1 | F13A1 | F5 | FBXL13 | FOCAD |
| FSIP1 | GADD45G | GALR3 | GLP1R | GLRA1 |
| GP9 | GREB1L | HBG2 | HPSE2 | IER3 |
| IGSF1 | IMPG2 | KCNH1 | KCNH7 | LHCGR |
| LRP1B | LUM | MED12L | OTOG | PCDHGA5 |
| PRAME | RIMS1 | RTEL1 | SCLT1 | SELL |
| SFN | SLC18A3 | SLC9B1 | TAC1 | TDO2 |
| TET1 | VCAN | WNT2 | ZBTB32 |  |

**Supplemental Table 2** The antibodies and catalog numbers used for western blot

| Antibody (Concentration) | Vendor | Catalog Number |
| --- | --- | --- |
| Versican (VCAN) (1:1000) | ABclonal | A19655 |
| TLR2 (1:1000) | ABclonal | A11225 |
| p-PI3K (1:1000) | ABclonal | AP0854 |
| p-AKT (1:1000) | ABclonal | AP0140 |
| FOSL1 (1:1000) | ABclonal | A21988 |
| FAS/CD95 (1:1000) | Proteintech | 60196-1-Ig |
| JUN (1:1000) | Proteintech | 24909-1-AP |
| PCNA (1:1000) | Proteintech | 60097-1-Ig |
| BCL2 (1:1000) | Proteintech | 12789-1-AP |
| ACTIN (1:100,000) | ABclonal | AC026 |

**Supplemental Figure 1** Intersection analysis between three gene groups of genes associated with cancer recurrence, primary/recurrence differential expression genes, and prognosis influenced genes, and pan-cancer recurrence related genes

**Supplemental Figure 2** GSEA functional enrichment and ESTIMATE regression analysis of recurrent-associated expressed molecules (RAEM). GSEA enrichment pathway shows **A,** ‘Cell Cycle’, **B,** ‘DNA Replication’, **C,** ‘MAPK Signaling Pathway’, **D,** ‘Notch Signaling Pathway’, **E,** ‘P53 Signaling Pathway’ **F,** ‘WNT Signaling Pathway’ are all significantly upregulated (FDR<0.25, P<0.05);


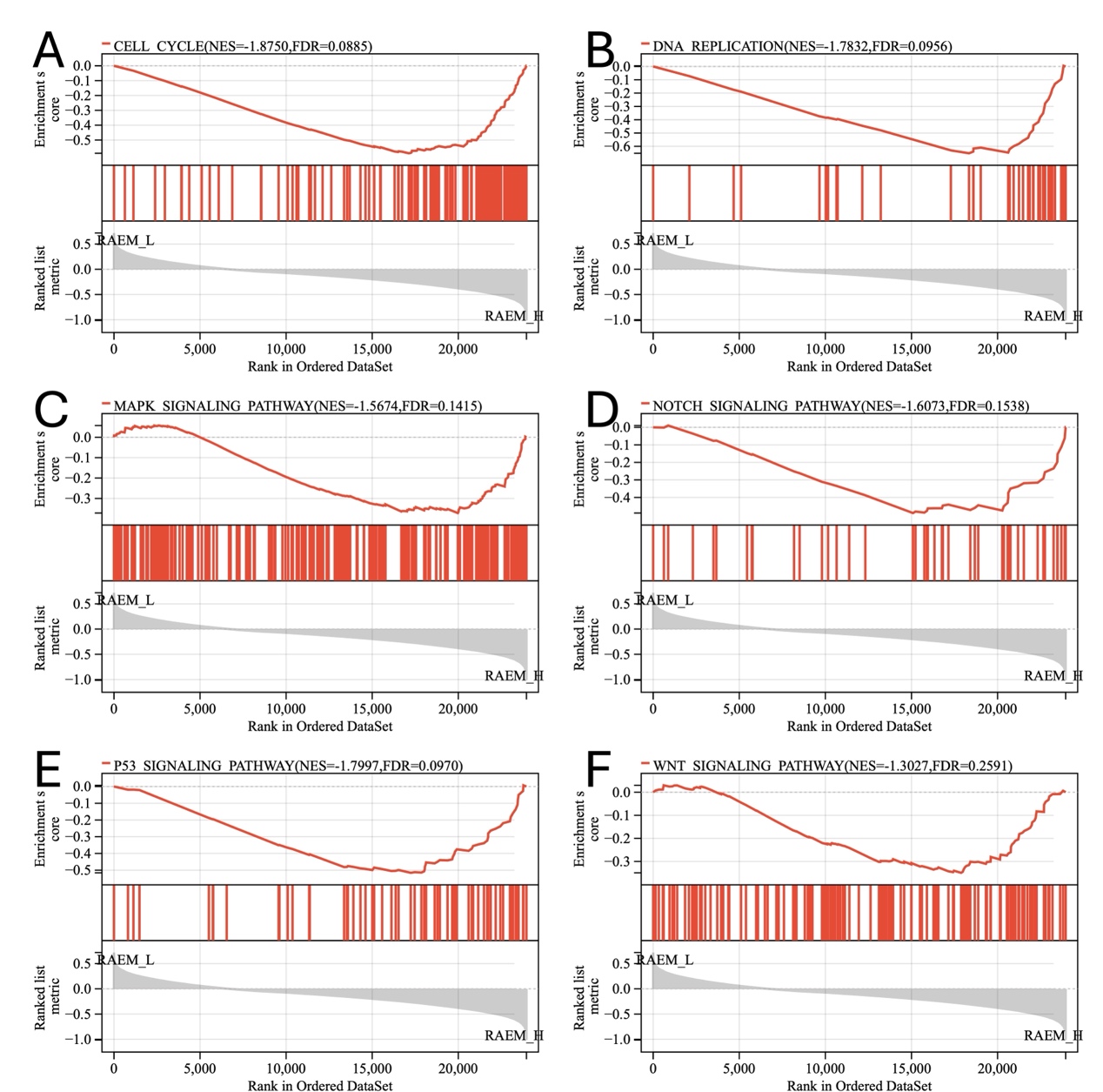


**Supplemental Table 3** Summary of clinical information of CGGA cohort

| **Characteristics** | **All Sample, N=657** |
| --- | --- |
| **Age, years** |  |
| Average ± SD | 43.25 ± 12.41 |
| **Gender, n (%)** |  |
| Female | 283 (43.1%) |
| Male | 374 (56.9%) |
| **WHO Grade, n (%)** |  |
| II | 172 (26.2%) |
| III | 248 (37.7%) |
| IV | 237 (36.1%) |
| **Histology, n (%)** |  |
| A (Astrocytoma) | 78 (11.9%) |
| AA (Anaplastic Astrocytoma) | 80 (12.2%) |
| AO (Anaplastic Oligodendrogliomas) | 46 (7.0%) |
| AOA (Anaplastic Oligodendroastrocytoma) | 15 (2.3%) |
| GBM (Glioblastoma) | 133 (20.2%) |
| O (Oligodendrogliomas) | 44 (6.7%) |
| OA (Oligodendroastrocytoma) | 8 (1.2%) |
| rA (recurrent Astrocytoma) | 27 (4.1%) |
| rAA (recurrent Anaplastic Astrocytoma) | 69 (10.5%) |
| rAO (recurrent Anaplastic Oligodendrogliomas) | 33 (5.0%) |
| rAOA (recurrent Anaplastic Oligodendroastrocytoma) | 5 (0.8%) |
| rGBM (recurrent Glioblastoma) | 104 (15.8%) |
| rO (recurrent Oligodendrogliomas) | 14 (2.1%) |
| rOA (recurrent Oligodendroastrocytoma) | 1 (0.2%) |
| **IDH, n (%)** |  |
| Mutant | 333 (54.7%) |
| Wild | 276 (45.3%) |
| **1p/19q, n (%)** |  |
| Co-deletion | 137 (23.2%) |
| Non co-deletion | 454 (76.8%) |
| **MGMTp methylation, n (%)** |  |
| Methylated | 304 (58.2%) |
| Un-methylated | 218 (41.8%) |
| **P/R State, n (%)** |  |
| Primary | 404 (61.5%) |
| Recurrent | 253 (38.5%) |
